# RAGE Up-Regulation Differently Affects Cell Proliferation and Migration in Pancreatic Cancer Cells

**DOI:** 10.3390/ijms21207723

**Published:** 2020-10-19

**Authors:** Priyanka Swami, Swetha Thiyagarajan, Arianna Vidger, Venkata S. K. Indurthi, Stefan W. Vetter, Estelle Leclerc

**Affiliations:** Department of Pharmaceutical Sciences, College of Health Professions, North Dakota State University, Fargo, ND 58105, USA; Priyanka.Swami@ndsu.edu (P.S.); swetha.thiyagarajan@ndsu.edu (S.T.); arianna.vidger@ndsu.edu (A.V.); shravan.indurthi@gmail.com (V.S.K.I.); Stefan.Vetter@ndsu.edu (S.W.V.)

**Keywords:** RAGE, cell proliferation, cell migration, pancreatic cancer cells

## Abstract

The receptor for advanced glycation end products (RAGE) contributes to many cellular aspects of pancreatic cancer including cell proliferation, migration, and survival. Studies have shown that RAGE activation by its ligands promotes pancreatic tumor growth by stimulating both cell proliferation and migration. In this study, we investigated the effect of RAGE up-regulation on the proliferation and migration of the human pancreatic cancer Panc-1 cell-line. We show that moderate overexpression of RAGE in Panc-1 cells results in increased cell proliferation, but decreased cell migration. The observed cellular changes were confirmed to be RAGE-specific and reversible by using RAGE-specific siRNAs and the small molecule RAGE inhibitor FPS-ZM1. At the molecular level, we show that RAGE up-regulation was associated with decreased activity of FAK, Akt, Erk1/2, and NF-κB signaling pathways and greatly reduced levels of α2 and β1 integrin expression, which is in agreement with the observed decreases in cell migration. We also demonstrate that RAGE up-regulation changes the expression of key molecular markers of epithelial-to-mesenchymal transition (EMT). Our results suggest that in the absence of stimulation by external ligands, RAGE up-regulation can differently modulate cell proliferation and migration in pancreatic cancer cells and regulates partly EMT.

## 1. Introduction

The receptor for advanced glycation end products (RAGE) is an immunoglobulin-like cell surface receptor involved in cancer, complications of diabetes, and neurodegenerative disorders [1,2,3,4]. RAGE is activated by several groups of unrelated ligands including advanced glycation end products (AGE), S100 proteins, amyloid β peptides, as well as DNA and glycosaminoglycans. RAGE is described as a receptor for damage-associated molecular patterns (DAMPs) because many RAGE ligands are associated with tissue damage, inflammatory or metabolic stress [5,6,7,8,9]. RAGE is normally expressed at low levels in most healthy tissues, exceptions being embryonic tissues and adult lung tissue [10]. However, RAGE expression is higher in diseased human tissues including tumors (brain, brain, colon, colorectal, lung, ovarian cancer, lymphoma, and melanoma) [4], as well as tissues and organs from patients with chronic inflammatory disorders (rheumatoid arthritis, arteriosclerosis, inflammatory bowel disease, and complications from diabetes) [11]. RAGE knock-out animals are viable and present a phenotype similar to that of wild-type animals besides having signs of mild hyperactivity and increased sensitivity to auditory stimuli [12]. However, in animal models, RAGE has been shown to accelerate the progression of many diseases such as sepsis and systemic bacterial infection, arthritis, multiple sclerosis, complications from diabetes, or cancer [13,14,15,16].

Typically, RAGE activation results from engagement by its ligands. RAGE activation can initiate multiple types of signaling pathways, depending on the ligand, its concentration, and the cell type where RAGE is activated. RAGE-dependent signaling cascades include MAP kinases (p38, c-Jun N-terminal kinase (JNK), and extracellular signal-related kinases (ERK1/2)), the PI3K/Akt pathway as well as the Janus kinase–signal transducer and activator of transcription (JAK-STAT) pathway [1,17,18,19]. Ultimately, RAGE signaling leads to the activation of several transcription factors including NF-κB, SP-1, and AP-1. This results in a positive feedback loop because RAGE expression itself is under the control of these transcription factors [17,20,21,22,23].

In cancer, RAGE has been shown to either promote or suppress tumor formation, depending on the type of tumor. For instance, RAGE has tumor-promoting activities in breast, colon, prostate, oral squamous cell cancer, melanoma, and lymphoma [4,24], but tumor-suppressing activities in lung cancer and rhabdomyosarcoma [25,26,27].

In mouse models of pancreatic ductal adenocarcinoma, it has been shown that RAGE promotes the development of pancreatic lesions and tumors. In these studies, cross-breeding of conditional KRAS^G12D/+^ mice, which spontaneously develop cancer lesions in their pancreas, with RAGE^−/−^ knock-out mice, resulted in fewer pancreatic lesions and longer survival rates than in KRAS^G12D/+^ RAGE^+/+^ mice [16,18]. In the study of Kang et al., the authors observed that RAGE protein levels progressively increased in the tumor tissues as pancreatic lesions progressed, suggesting a role of RAGE, not only in the initiation of PDAC, but also in the progression of the disease. In addition, in these tumors, RAGE was found overexpressed in the cancer lesions but not in adjacent normal tissue [18].

Two ligands of RAGE, S100P, and high mobility group box 1 (HMGB1), have been extensively studied in the context of pancreatic cancer. S100P stimulates both cell proliferation and migration of human pancreatic cancer Panc-1 cells, in a RAGE dependent manner [28]. S100P has also been shown to protect Panc-1 cells against the cytotoxicity of 5-fluorouracil [28]. Studies have shown that S100P overexpressing Panc-1 cells grew five-fold larger subcutaneous tumors than controls and that inhibition of the S100P/RAGE interaction could inhibit tumor growth [28,29,30,31,32,33]. Other studies by Kang et al. showed that activation of RAGE by HMGB1 stimulated tumor growth, and favored tumor cell survival by increasing autophagy and reducing apoptosis [18,34,35,36,37,38].

Besides these reports, no study has yet investigated the effect of RAGE up-regulation on cell proliferation and cell migration of pancreatic cancer cells, in the absence of stimulation by external ligands. The aim of the present study was to fill this gap in knowledge. To study the effect of RAGE up-regulation, we compared the cellular behavior of the parental human pancreatic cancer Panc-1 cell-line with that of two daughter cell-lines (named Full-length RAGE 2 and 3 Panc-1 cells (FLR2, and FLR3)), which stably overexpress RAGE. We show that in Panc-1 cells, in the absence of stimulating ligands, RAGE overexpression results in increased cell proliferation, but in decreased cell migration. We show that these changes in cell proliferation and migration are reversible, using RAGE targeting siRNAs. Importantly, the changes in cell proliferation and migration could also be reversed using the small molecule RAGE inhibitor FPS-ZM1. The decreases in cell migration observed in RAGE expressing FLR2 and FLR3 cells were associated with decreased activation of focal adhesion kinase (FAK), ERK1/2, Akt, and NF-κB and with suppression of α2 and β1 integrin expression. In addition, changes in EMT marker levels were also observed in RAGE overexpressing cells.

Overall, our study demonstrates that in the absence of stimulation by external ligands, RAGE up-regulation is sufficient to alter cellular behavior, and results in opposite effects on cell proliferation and migration in human Panc-1 pancreatic cancer cells. Our data suggest that RAGE could have intricate functions in pancreatic cancer depending on its expression level in tumor tissues.

## 2. Results and Discussion

### 2.1. Generation and Characterization of RAGE Overexpressing Panc-1 Cells

The goal of our study was to determine the effect of RAGE up-regulation in the human Panc-1 pancreatic cancer cell-line. Two RAGE overexpressing Panc-1 cell-lines were generated, named FLR2 and FLR3 Panc-1. RT-PCR analysis showed that FLR2 and FLR3 Panc-1 cells synthesized 13.4 +/− 1.3- and 14.2 +/− 1.1-fold higher transcript levels of RAGE, respectively, than WT Panc-1 cells. As the level of RAGE in the parental Panc-1 cell-line was too low to be detected by Western blot, we used quantitative ELISA to compare RAGE protein levels in the parent and the two daughter cell-lines. We calculated that WT Panc-1 cells contained 12.1 +/− 1.1 pg RAGE per mg of total protein. The level of RAGE in FLR2 and FLR3 Panc-1 cells was 5.4-fold (65.3 +/− 0.1 pg) and 4.4-fold (53.5 +/− 10.1 pg), respectively, higher than that in WT Panc-1 cells. Although moderate, this level of RAGE up-regulation in FLR2 and FLR3 cells could mimic the up-regulation of RAGE observed in pancreatic tumor lesions during tumor progression [17].

### 2.2. Effect of RAGE Up-Regulation on Cell Proliferation

**Figure 1:**
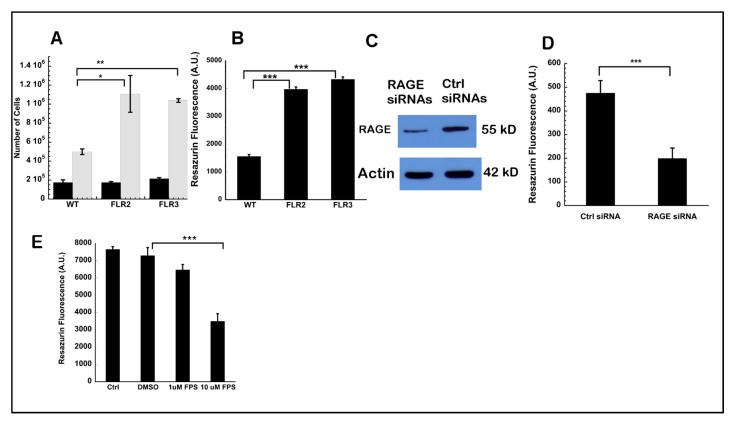
(**A**,**B**) RAGE expressing FLR2 and FLR3 Panc-1 cells have higher proliferation rates than WT Panc-1 cells. (**A**) Cell proliferation was assessed by Trypan blue exclusion assay. Black bars: number of cells at the time of seeding; gray bars: number of cells after a 48 h incubation. (**B**) Cell proliferation was assessed using resazurin fluorescence 48 h after cell seeding. (**C**) RAGE silencing reduces RAGE expression levels in FLR2 Panc-1 cells. FLR2 Panc-1 cells were transfected using RAGE specific siRNAs or control (Ctrl) siRNAs. (**D**) RAGE silencing reduces the proliferation of FLR2 Panc-1 cells. FLR2 Panc-1 cells were silenced with either RAGE specific siRNAs (RAGE siRNA) and scrambled siRNAs (Ctrl siRNAs). (**E**) RAGE inhibition reduces the proliferation of FLR2 Panc-1 cells. Cell proliferation was assessed using resazurin fluorescence. Cells were incubated with media only (Ctrl), 0.2% DMSO, 1 µM or 10 µM FPS-ZM1. * *p* < 0.05; ** *p* < 0.01; *** *p* < 0.001.

We first investigated whether RAGE overexpression influenced cell proliferation using two complementary methods: Trypan blue exclusion assay and resazurin proliferation assay. The Trypan blue exclusion assay showed a 2.2-fold and a 1.7-fold higher number of cells in FLR2 and FLR3 Panc-1 cells respectively, after a 48 h incubation, compared to WT Panc-1 cells (Figure 1A). Similar observations were made using the metabolic dye resazurin, with a 2.5- and 2.8-fold increase in resazurin fluorescence in FLR2 and FLR3 Panc-1 cells, respectively, compared to WT Panc-1 cells (Figure 1B). Our data show that RAGE up-regulation in Panc-1 cells increases the rate of cell proliferation. We next investigated if silencing RAGE using siRNAs or blocking RAGE activation using a small molecule inhibitor could reverse the effect of RAGE up-regulation. As both FLR2 and FLR3 behave similarly in terms of cell proliferation, we performed the next assays with only the FLR2 clone.

FLR2 Panc-1 cells were transfected either with a mixture of three RAGE specific siRNAs or with scrambled siRNAs as controls. To assess the efficiency of silencing with the RAGE siRNAs, we performed Western blot analysis and determined that RAGE expression was reduced by 60% in the presence of RAGE siRNAs, (Figure 1C). In the cell proliferation assay, we observed a 2.4-fold decrease in the proliferation rate of FLR2 after transfection with RAGE specific siRNAs, compared to cells transfected with scrambled siRNAs (Figure 1D). These data demonstrate that the observed effect on cell proliferation was clearly linked to RAGE expression.

We next investigated if the RAGE-dependent increases in cell proliferation could be reversed using a small molecule inhibitor of RAGE, FPS-ZM1. Treatment with 1 µM FPS-ZM1 showed a modest but non-significant decrease in cell proliferation, however, this effect was more pronounced (2.1-fold reduction), and statistically significant when 10 µM FPS-ZM1 were used (Figure 1E). FPS-ZM1 is a small molecule that binds to the V-domain of RAGE and has been shown to inhibit the interaction of RAGE with ligands binding to its V-domain [39,40]. However, because the V-domain might be involved in RAGE dimerization and signaling [41], FPS-ZM1 might also affect RAGE signaling, in the absence of ligands. Taken together, our data strongly suggest that RAGE up-regulation in Panc-1 cells increases cell proliferation.

### 2.3. Effect of RAGE Up-Regulation on Cell Migration

**Figure 2:**
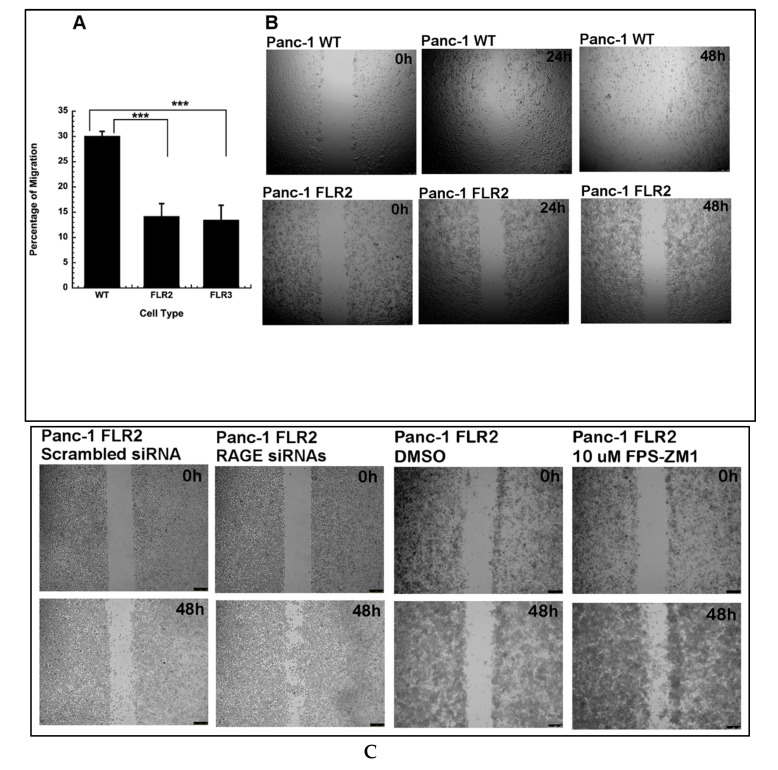
(**A**,**B**) RAGE expression decreases cell migration in FLR2 Panc-1 cells. Cell migration was assessed using the Boyden chamber migration assay. The percentage of migrated cells 24 h after seeding was estimated using resazurin. (**B**) RAGE expression reduces wound healing in FLR2 Panc-1 cells assay with WT and FLR2 Panc-1 cells. Cells were images at 0 h, 24 h, and 48 h following the formation of the wound. Representative images are shown. (**C**) RAGE silencing in FLR2 Panc-1 cells reverses wound healing inhibition in FLR2 Panc-1 cells. Wound healing assay with FLR2 Panc-1 cells that had been either transfected with RAGE specific siRNAs or scrambled siRNAs, or treated with either vehicle (0.2% DMSO) or 10 µM FPS-ZM1. Images were taken at t = 0 h and t = 48 h. Representative images are shown. *** *p* < 0.001.

Studies from Arumugam et al. showed that activation of RAGE by S100P in Panc-1 cells increased both cell proliferation and migration [28,29,30,31,32,33]. Based on these reports, we hypothesized that RAGE expression in Panc-1 cells would increase both cell proliferation and migration. Two different assays were used to assess cell migration: the Boyden chamber cell migration assay and the wound healing assay. Using the Boyden chamber assay, we observed that after a 24 h incubation, 2.1-fold fewer FLR2 (14.2% +/− 2.5%) and 2.2-fold fewer FLR3 (13.5% +/− 2.9%) Panc-1 cells had migrated through the filter than WT Panc-1 cells (30.1% +/− 0.95%) (Figure 2A). In the wound healing assay, we observed that WT Panc-1 cells had completely covered the wounded area after a 24 h incubation, whereas FLR2 Panc-1 cells did not show significant coverage of this area, neither after a 24 h nor 48 h incubation (Figure 2B). Similar results were observed with FLR3 Panc-1 cells (data not shown). The results of the cell migration and wound healing assays suggest that FLR2 Panc-1 cells are defective in migration compared to the parental Panc-1 cells. To demonstrate that RAGE was responsible for the observed change in cell migration, we performed the wound healing assay after silencing RAGE in FLR2 Panc-1 cells using specific siRNAs, scrambled siRNAs were used as controls. The data show that silencing RAGE in FLR2 Panc-1 cells restored cells’ abilities to migrate to the same extent as WT Panc-1 cells (Figure 2C).

As expected, FLR2 Panc-1 cells transfected with scrambled siRNAs did not regain their migration abilities. To further demonstrate that RAGE was responsible for the changes in cell migration, we performed the wound healing assay in the presence of the small molecule RAGE inhibitor FPS-ZM1. After a 48 h treatment with FPS-ZM1, we observed significant differences in the wound area coverage when compared to FLR2 Panc-1 cells treated with a vehicle (0.2% DMSO) (Figure 2C).

Our data appear to contradict those reported by Arumugam et al. where RAGE activation by S100P increased both cell proliferation and migration of Panc-1 cells [28,29,30,31,32,33]. However, this might be explained by differences in the experimental conditions. In our study, RAGE is overexpressed in Panc-1 cells, and no ligand is added, whereas in the study of Arumugam et al., Panc-1 cells were stimulated with S100P. Although S100P does interact with RAGE, it also interacts with additional target proteins modulating cell migration, such as the scaffolding protein IQGAP1, the cytoskeletal associated protein ezrin, or the S100P binding protein (S100PBP) (reviewed in [42]). These additional interactions might explain the overall increase in cell migration observed with S100P.

### 2.4. The Levels of α2 and β1 Integrins Are Significantly Decreased in RAGE Overexpressing Cells

**Figure 3:**
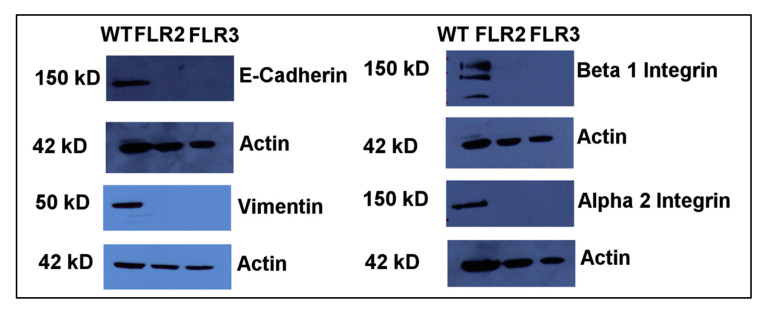
Expression levels of E-cadherin, vimentin, and α2 and β1 integrins are reduced in FLR2 and FLR3 Panc-1 cells as compared to WT Panc-1 cells. Protein expression levels in WT, FLR2, and FLR3 Panc-1 cells were determined by Western blot. Actin was used as a loading control. Representative blots are shown.

The decreases in cell migration in RAGE overexpressing Panc-1 cells led us to investigate the expression levels of α2 and β1 integrins in WT, FLR2, and FLR3 Panc-1 cells. Integrins form heterodimeric cell surface receptors that mediate interactions with extracellular matrix proteins and control cellular functions such as adhesion, proliferation, migration as well as cell survival [43,44]. Among the different integrins, the heterodimer α2/β1 has been shown to mediate a malignant phenotype, by increasing adhesion, proliferation, and migration, in a broad panel of human pancreatic cancer cell-lines [45,46,47]. We analyzed the transcript levels of α2 and β1 integrins in WT, FLR-2, and FLR-3 Panc-1 cells by RT-PCR. We were able to detect α2 integrin transcripts in WT Panc-1 cells (∆Ct (Ct_integrin_ − Ct_actin_ = 11.37 +/− 0.26)) but not in FLR2 and FLR3 Panc-1 cells (no Ct value for α2 integrin was observed in these cells). Similarly, significantly higher levels of β1 integrin transcripts were detected in WT Panc-1 cells = 6.51 +/− 0.29) than in FLR2 (∆Ct = 15.12 +/− 0.57) and FLR3 (∆Ct = 15.50 +/− 0.81) Panc-1 cells. A large difference in transcript levels of α2 and β1 integrins observed between WT, and FLR2 and FLR3 cells was also observed at the protein level, as shown by Western blot analysis. Whereas α2 integrin was detected by Western blot in cell extracts from WT Panc-1 cells, no band was detected for FLR2 and FLR3 Panc-1 cells (Figure 3). Similarly, bands corresponding to β1 integrin were visible only in WT Panc-1 cells; no band could be detected for FLR2 and FLR3 Panc-1 cells (Figure 3). Additional investigations by flow cytometry also confirmed the difference in the expression levels of α2 and β1 integrins between WT and FLR2 Panc-1 cells.

**Figure 4:**
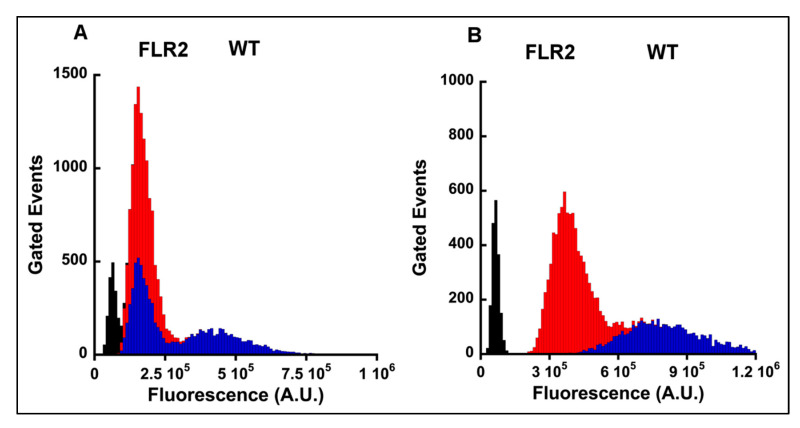
Lower expression levels of α2 and β1 integrins are observed in FLR2 Panc-1 cells than in WT Panc-1 cells. Expression levels of α2 (**A**) and β1 integrins (**B**) in FLR2 and WT Panc-1 cells were determined by flow cytometry. Staining for α2 or β1 integrin in WT Panc-1 cells is shown as blue histograms, and in FLR2 Panc-1 cells as red histograms. Staining of cells with the secondary antibody only is indicated by the black histogram in each figure. The experiments were performed three independent times. Representative histograms are shown.

Staining of WT Panc-1 cells for α2 integrin revealed two populations of cells with different α2 integrin expression levels: one population showed a mean fluorescence intensity peak at 4 × 10^5^ A.U., while the second population displayed a fluorescence peak at 1.3 × 10^5^ A.U. The presence of multiple cell populations in cancer cell-lines is not unusual due to the inherent heterogeneity of cancer cells in terms of chromosome number. For instance, as reported on the ATCC website, 22% Panc-1 cells in culture possess 63 chromosomes. Therefore, differences in chromosome number between individual cells within the populations of Panc-1 cells could explain the different levels of α2 integrin expression. Not surprisingly, in FLR2 Panc-1 cells, only one population of cells was observed when assayed for α2 integrin expression. Only one population is observed because α2 integrin expression is suppressed in the FLR2 Panc-1 cells (Figure 4A). Although it is possible that a population of cells with low levels of α2 integrin was selected during the cloning process, this is not likely, because if this were the case, then we would not observe changes in migration in these cells when RAGE is silenced with siRNAs.

In WT Panc-1 cells analyzed for β1 integrin, only a single population of cells was observed with a fluorescence peak of 7.5 × 10^5^ A.U., whereas a cell population with a lower fluorescence (4 × 10^5^ A.U.) peak was observed in FLR2 Panc-1 cells (Figure 4B). Overall, the flow cytometry data are in agreement with the data obtained by Western blot and provide an explanation for the reduced migration properties of FLR2 Panc-1 cells.

### 2.5. RAGE Overexpression Is Associated with Changes in Expression Levels of Vimentin and E-Cadherin

We next investigated how RAGE overexpression affected the levels of E-cadherin and vimentin as these two proteins have important functions in both proliferation and migration. Vimentin is an intermediate filament that interacts with α2/β1 integrins, and its expression correlates with increased invasion and migration in cancer cells [48,49]. Vimentin allows epithelial cells to increase their invasion properties [50]. We observed lower levels of vimentin in FLR2 and FLR3 Panc-1 cells compared to WT Panc-1 cells (Figure 3), in agreement with the lower levels of α2/β1 integrins and the reduced migratory abilities of these cells.

E-cadherin is a cell adhesion molecule that promotes cell–cell interactions, allows cohesion between cells and tissue integrity [51]. During cell migration of cancer cells, E-cadherin is often down-regulated, reducing cell–cell interactions in favor of cell–ECM interactions and allowing cells to migrate and invade the stroma [50]. Besides its function on cell adhesion, E-cadherin can also influence cellular signaling by transducing growth-inhibitory signals [52,53,54,55], and negatively regulating cell proliferation [56]. In pancreatic cancer cells, disruption of E-cadherin-mediated cell–cell interactions has been shown to promote cell proliferation [57]. We observed lower levels of E-cadherin in FLR2 and FLR3 than in WT Panc-1 cells. The increase in proliferation in FLR2 and FLR3 cells could thus be correlated with the down-regulation of E-cadherin (Figure 3).

**Figure 5:**
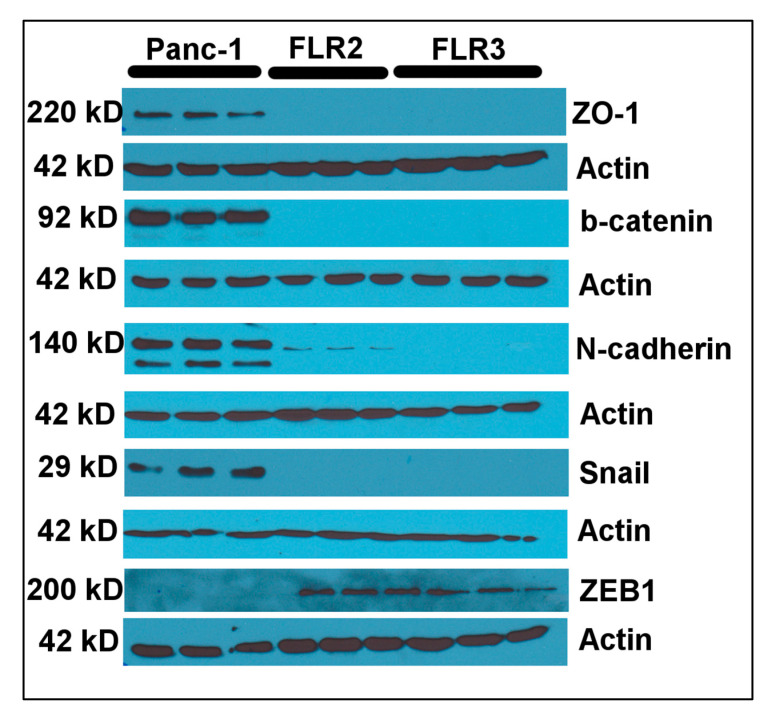
RAGE overexpression results in the reprogramming of several EMT markers. Protein expression levels of five epithelial or mesenchymal markers (ZO-1, β-catenin, N-cadherin, snail, and ZEB1) in three independent cell lysates of WT, FLR2, and FLR3 Panc-1 cells were determined by Western blot. Actin was used as a loading control.

As previously reported by others, and as shown in our study, Panc-1 cells have a “mixed” phenotype in terms of epithelial and mesenchymal marker expression, with the presence of both E-cadherin (epithelial) and vimentin (mesenchymal) [58]. We further investigated if other markers of epithelial-to-mesenchymal transition (EMT) were also modulated by RAGE overexpression. In our experimental conditions, we detected zona occludens protein 1 (ZO-1) (Figure 5) in addition to E-cadherin in Panc-1 cells (Figure 5). ZO-1 is found in tight junctions whereas E-cadherin is found in adherens junctions [59]. Adherens and tight junctions are essential for the maintenance of epithelial cell homeostasis through the formation of strong cell–cell interactions [60]. During EMT, adherens and tight junctions are weakened and disassembled due to the loss of E-cadherin and increase of N-cadherin expression. When adherens junctions disassemble, β-catenin, that is bound to the cytoplasmic domain of E-cadherin, migrates to the nucleus and acts as a transcriptional co-activator of EMT inducer genes, such as Snail (*SNAI1*) and ZEB1 [61]. β-catenin was found expressed in Panc-1 cells (Figure 5), in addition to the mesenchymal marker N-cadherin, further supporting the presence of a mixed epithelial/mesenchymal phenotype in Panc-1 cells. We also detected the EMT-inducer and transcription factor Snail in Panc-1 cells, but could not detect, in our experimental conditions, the other EMT-inducer and transcription factors ZEB1 (Figure 5) and Slug (*SNAI2*) (data not shown).

Analysis of the Panc-1 FLR2 and FLR3 cell lysates by Western blot showed the absence of N-cadherin, β-catenin, and Snail in these cells (Figure 5). Components of the adherens junctions (E-cadherin) and tight junctions (ZO-1) were also absent (Figure 3 and Figure 5). However, we could detect ZEB1 in these cells (Figure 5). Our data suggest that a significant reprogramming occurs in Panc-1 cells upon RAGE overexpression. Several epithelial and mesenchymal markers become down-regulated, while at least one EMT-inducer protein, ZEB1, is up-regulated. The outcomes of cell proliferation and migration assays strongly suggest that these changes in gene expression affect mainly the mobility of Panc-1 cells. Additional studies will be necessary to provide additional details on the EMT related signaling pathways affected by RAGE overexpression in Panc-1 cells.

### 2.6. Overexpression of RAGE Results in Down-Regulation of FAK, ERK1/2, Akt, and NF-κB

**Figure 6:**
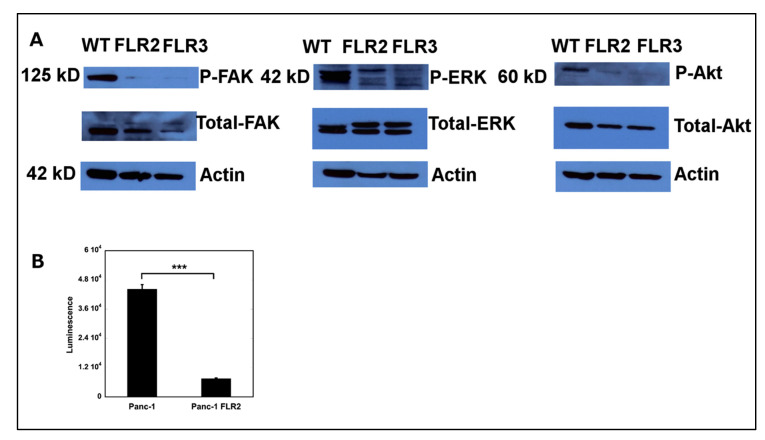
(**A**) RAGE expression in FLR2 Panc-1 cells affects cell signaling. Expression levels of ERK1/2 and pERK1/2, Akt and P-Akt, FAK, and P-FAK in WT, FLR2, and FLR3 Panc-1 cells as determined by Western blot analysis. Actin was used as a loading control. Representative blots are shown. (**B**) RAGE expression in FLR2 Panc-1 cells results in decreased NK-κB activity. NK-κB activity assay using an NF-κB luciferase reporter plasmid. NF-κB activity is reported as arbitrary luminescence units. A representative assay is shown. *** *p* < 0.001.

We next investigated how RAGE overexpression affected signaling pathways downstream of integrins. Focal adhesion kinase (FAK) is a cytoplasmic tyrosine kinase that transduces signals from integrins, through both outside-in and inside-out processes [62,63,64,65,66]. FAK influences cell motility, invasion, survival, and proliferation [66]. Both up-regulation and increased activity of FAK have been previously reported in metastatic diseases [67]. We found lower levels of both phosphorylated and non-phosphorylated forms of FAK, in FLR2 and FLR3 compared to WT Panc-1 cells (Figure 6A). As FAK signals through ERK and Akt [66], we also measured the levels of phosphorylated and total Akt and ERK1/2 in the WT, FLR2, and FLR3 Panc-1 cells. In agreement with the lower levels of FAK activity, we found lower phosphorylation levels of Akt and ERK1/2 in Panc-1 FLR2 and FLR3 than in wild type Panc-1 cells (Figure 6A).

ERK1/2 and Akt signaling cascades are involved in both cell proliferation and migration [68]. Both kinases phosphorylate more than 200 different substrates including membrane-associated proteins, cytosolic cytoskeletal elements, and nuclear proteins [68,69]. Three isoforms of Akt (Akt 1, 2, and Akt3) have been described in human cells [70]. The role of Akt1 and Akt2 has been extensively studied in breast cancer cells and mouse models. Studies have shown that Akt1 and Akt2 can have opposing effects on cell migration and invasion: Akt1 was found to suppress migration and invasion while Akt2 promoted migration and invasion of breast cancer cells. Similarly, in a mouse model of breast cancer, Akt1 was shown to decrease metastasis while increasing tumorigenesis and Akt2 increased tumor metastasis [71,72,73,74,75]. In our study, we used a pan-Akt antibody which recognizes all three Akt isoforms and we could not further detail which Akt isoform was downregulated upon RAGE overexpression. Further studies using isoform-specific antibodies will be necessary to identify the Akt isoforms that are specifically modulated by RAGE overexpression in Panc-1 cells. This knowledge could lead to a better understanding of how RAGE triggers cell-type specific effects.

One common transcription factor downstream to both the ERK1/2 and Akt pathways is NF-κB. NF-κB regulates cell proliferation, cell migration and invasion, and tumorigenesis [76,77]. We observed a six-fold decrease in NF-κB activity in FLR2 Panc-1 cells compared to WT Panc-1 cells. This decrease in activity appears to be positively correlated with the decrease in cell proliferation as well as the decrease in ERK1/2 and Akt activation (Figure 6B).

## 3. Materials and Methods

### 3.1. Cell Culture

Human Panc-1 pancreatic cancer cells were purchased from the American Type Culture Collection (ATCC, Manassas, VA, USA). Cells were grown and maintained in DMEM supplemented with 10% FBS (ATCC), penicillin (100 U/mL), and streptomycin (100 μg/mL) (GE Healthcare Life Science, Pittsburg, PA, USA). RAGE overexpressing FLR2 and FLR3 Panc-1 cells were maintained in the same medium supplemented with 0.25 mg/mL G418 (Corning Incorporated, Corning, NY, USA). Depending on the cell-based assay, cells were detached either mechanically using a cell scraper or following incubation with 0.05% trypsin/EDTA (GE Healthcare Life Science). Cell counting was performed manually using a hematocytometer.

### 3.2. Generation of Stably Transfected FLR2 and FLR3 Panc-1 Cells

FLR2 and FLR3 Panc-1 cells were generated by stably transfecting Panc-1 cells with a pcDNA3 plasmid coding for full-length RAGE (NM_001136) (pcDNA3-FLRAGE, kindly provided by Dr. C.W. Heizmann, Children’s Hospital, Zurich, Switzerland). For the transfection, cells were seeded at 50,000 cells per well in a 24-well plate. Following attachment, the cells were transfected with pcDNA3-FLRAGE using Lipofectamine 2000 according to the recommendations of the manufacturer (Invitrogen/ThermoFisher Scientific, Waltham, MA, USA). Transfected cells were grown in cell culture media containing 0.25 mg/mL G418 and individual clones were selected after diluting the cell suspension to a single cell per well. Two independent clones, FLR2 Panc-1 and FLR3 Panc-1 were isolated through this selection process.

### 3.3. Determination of RAGE Transcript and Protein Levels in FLR2 and FLR3 Panc-1 Cells

RAGE transcript levels were determined by real-time (RT) PCR. Total RNAs were extracted using the PARIS kit (Invitrogen/ThermoFisher Scientific, Waltham, MA, USA) according to the manufacturer’s instructions. The quality of the RNAs was assessed by absorbance spectroscopy and by agarose gel electrophoresis. RNAs were reverse transcribed into cDNA using the Reverse Transcription System from Promega (Madison, WI, USA).

RT-PCR was run using 10 ng cDNA per 20 μL sample, and Eva Green qPCR mix (Mango Technologies, Mountain View, CA, USA) on a Stratagene Mx3000p thermocycler. The β-actin gene was used as a housekeeping gene. The sequence of the primers used by RT-PCR is described in Table 1.

The following RT-PCR program was used: 5 min at 95 °C followed by 40 cycles of 15 s at 95 °C, 20 s at 60 °C, 20 s at 72 °C. A melting curve was recorded at the end of the 40 cycles to evaluate the quality of the amplified products. The fold (F) of change in gene expression was calculated for each gene using the Δ∆Ct method with F = 2^(∆∆Ct)^ and ∆∆Ct = ∆Ct_RAGE_ − ∆Ct_WT_. ∆Ct = Ct_gene_ − Ct_actin_ for Panc-1 cells overexpressing RAGE (∆Ct_RAGE_) and control wild-type (WT) Panc-1 cells (∆Ct_WT_). The experiments were run in triplicate using cDNAs obtained from three independent RNA preparations. The standard deviations were calculated for the magnitude of changes in gene expression.

### 3.4. Western Blot Detection of Akt/P-Akt, ERK/P-ERK, FAK/P-FAK, Vimentin, E-Cadherin, N-Cadherin, β-Catenin, Snail, Slug, ZEB1, ZO-1, α2 and β1 Integrins, and Actin

Cell extracts were prepared using the PARIS kit following the instructions recommended by the manufacturer. Proteins (25–50 μg) were resolved on 10 or 15% SDS PAGE and blotted onto nitrocellulose membranes according to standard procedures. The blots were blocked with either 5% milk powder or 3–5% BSA in Tris-buffered saline (TBS) at pH 7.4. The primary antibodies (Table 2) were diluted into 1 to 3% BSA in TBS containing 0.1% tween 20 (TBS-T), or 5% milk powder, according to the recommendations of the provider. HRP conjugated secondary antibodies (Table 2) (donkey anti-rabbit, goat anti-mouse, or rabbit anti-goat) were all from Jackson ImmunoResearch (West Grove, PA, USA) and were used at dilutions recommended by the manufacturer. Following the detection of the proteins of interest, the blots were stripped and incubated with a β-actin antibody. The blots were developed using a chemoluminescent substrate (Pierce ECL Western Blotting Substrate, ThermoScientific, Waltham, MA, USA). The signals were detected on autoradiography films using an X-ray film developer. The Western blots were performed with at least three independent cell extracts. Representative blots are shown in the figures.

### 3.5. Flow Cytometry Studies

WT and FLR2 Panc-1 cells (100,000 per 200 μL) were transferred to the wells of 96-well round bottom plates. The cells were fixed with 4% paraformaldehyde for 10 min, permeabilized with 0.3% Triton X-100 for 15 min on ice and washed three times with PBS containing 2% FBS. Cells were incubated with either β1 or α2 integrin antibody for 1 h on ice, washed three times with PBS containing 2% FBS, and incubated with AlexaFluor 488 conjugated secondary antibody for 30 min. See Table 2 for details about the antibodies used in the study. Cells were washed three times with PBS containing 2% FBS before being analyzed in a BD Accuri C6 Plus flow cytometer. For analysis, 10,000 healthy cells were gated based on their scattering (side- and forward-scattering), and cell fluorescence was detected at 488 nm (FL1-A). Experiments were performed three independent times. Representative histograms are shown in the figures.

### 3.6. Quantitative ELISA

For the quantitative ELISA, cell lysates were prepared using the PARIS kit cell lysis buffer. The protein level from each cell lysate was determined using the bicinchoninic assay (BCA, Pierce). RAGE levels were then determined using the Quantikine human RAGE Immunoassay kit (R&D Systems). A standard curve generated with a series of RAGE concentrations ranging from 20 pg/mL to 15,000 pg/mL was used to quantify RAGE levels for each cell lysate. RAGE protein levels were expressed in picogram per mg of total protein present in the cell extracts. The determination of RAGE by this method was performed three independent times in duplicate.

### 3.7. Cell Proliferation Assays

Two complementary methods were used to assess cell proliferation. In the first method, viable cells were counted after excluding dead cells using Trypan blue (Corning/VWR, Chicago, IL, USA). For this assay, 200,000 WT Panc-1, FLR2, or FLR3 cells were seeded in each well (2 mL) of a six-well plate. The cells were detached with trypsin after 48 h and viable cells were counted in triplicate. In the second assay, cell proliferation was assessed by measuring the changes in fluorescence of resazurin (Sigma Aldrich, St. Louis, MO, USA). The fluorescence properties of resazurin change upon reduction by reducing agents generated by viable cells. The resazurin assay was performed in 24-well plates using 40,000 cells per well. Resazurin (10 μg/mL) was added in each well 48 h after seeding and incubated for 3 h at 37 °C. Differences in cell proliferation were assessed by differences in fluorescence emission (Em: 590 nm) using an excitation wavelength of 540 nm. Cell proliferation assays of FLR2 Panc-1 cells were also performed in the presence of the small molecule RAGE inhibitor FPS-ZM1 (SelleckChem, Houston, TX, USA) as follows: wells of a 24-well plate were seeded with 40,000 cells per well (500 μL). Twenty-four hours after seeding, cell culture media was replaced with fresh media containing 1 μM or 10 μM FPS-ZM1 in the presence of 0.2% DMSO, and cells were incubated an additional 48 h. Control wells contained cell media and 0.2% DMSO only. Measurements of cell proliferation with resazurin were then performed as described above. All measurements were performed in triplicate. Both assays were performed three independent times.

### 3.8. Boyden Chamber Assay

Differences in cell migration between the different cell-lines were assessed using the Boyden chamber and the wound healing assays. In the Boyden chamber assay, cells (5 × 10^4^ cells/well) were seeded on top of 8 μm filter inserts (24-well plate, Greiner Bio-One) that were inserted into the wells of 24-well plates. DMEM supplemented with 10% FBS was added to the bottom of the well. After a 24 h incubation at 37 °C, cells that remained on top of the filter were gently removed using cotton swabs. Cells that had migrated through the filter remained attached to the other side of the insert. The filters were then re-inserted into their wells, which were supplemented with 10 g/mL resazurin. The fluorescence intensity (Ex: 540 nm; Em: 590 nm) was measured after a 4 h incubation. Wells containing cell culture media only were used as background. Wells seeded with cells in the absence of inserts were used as positive controls and the fluorescence values obtained from these wells were set to 100%. The percentage of migrated cells was calculated from the ratio R:

R = (Resazurin signal with insert − Resazurin background signal)/(Resazurin signal without insert − Resazurin background signal)

### 3.9. Wound Healing Assay

For this assay, a wound was created within a layer of confluent cells using a 20 μL pipet tip after creating the wound, the cell culture media was replaced with fresh media to remove detached cells. Images of the wound were taken at the time of the wound formation (t = 0 h) and after a 24 h or 48 h incubation. The experiments were performed in duplicate. This assay was also performed with cells transfected with RAGE targeting siRNAs or scrambled siRNAs, as well as in the presence of the small molecule inhibitor FPS-ZM1 (1 μM and 10 μM).

### 3.10. NF-κB Transcription Factor Activation Assay

For this assay, 35,000 cells were seeded in the wells of a 48-well plate for 24 h. The cells were then transfected with an NF-κB reporter plasmid coding for luciferase (NF-κB cis-Reporting System (Stratagene)) using lipofectamine 3000 (Invitrogen/Life Technologies) for 24 h. After transfection, the cells were lysed using the recommended lysis buffer (E1500, Promega, Madison, WI, USA). Luciferase activity was determined by luminescence using a luciferase substrate (E1500, Promega) on a Quik-Pak Quantifier luminometer (Berthold technologies, Pforzheim, Germany). The experiment was performed three independent times.

### 3.11. Transfection with RAGE siRNAs

Cells were seeded at 2 × 10^5^ cells/well in a six-well plate in the presence of a 2 mL antibiotic-free growth medium supplemented with 10% FBS. Transfection was performed using either RAGE specific siRNAs (sc-36374) or a scrambled control siRNA (sc-37007, Santa Cruz Biotechnologies, Dallas, TX, USA) using the instructions of the manufacturer. RAGE specific siRNAs consisted of a pool of three RAGE specific 19–25 nt siRNAs. Seventy-two hours after transfection, the cells were detached with a scraper, counted, and seeded for the resazurin cell proliferation assay and the wound healing assays, as described above. Three independent transfections were performed.

### 3.12. Statistical Analysis

The determination of the RAGE transcript levels in WT, FLR2, and FLR3 Panc-1 cells was performed in triplicate using cDNAs obtained from at least three independent RNA preparations. The Western blots for the detection of RAGE, Akt/P-Akt, ERK/P-ERK; FAK/P-FAK, vimentin, E-cadherin, β1 and α2 integrins, and actin were performed from three independent cell extracts. Representative blots are shown in Figure 1C, Figure 3 and Figure 5. Cell proliferation experiments were performed at least three times using six replicates per condition. Wound healing and migration assays were performed three times using three replicates per condition. The NF-κB activation assay was performed three independent times using three replicates.

Data are presented as means ± standard deviations (SD). Statistical analysis was performed by using the Student’s *t*-test. A *p*-value of less than 0.05 was considered as statistically significant. * *p* < 0.05; ** *p* < 0.01; *** *p* < 0.001.

## 4. Conclusions

Our study shows that RAGE up-regulation increases cell proliferation and reduces the migration properties of the human pancreatic cancer Panc-1 cell-line. The effects of RAGE up-regulation on cell proliferation and migration have been previously studied in several types of cancer and were shown to be dependent on the cancer cell type. For example, Kuniyasu et al. reported that RAGE silencing in the MKN28 gastric cancer cell-line did not affect cell proliferation in vitro, but did result in decreased cell invasion and migration [78]. In the WM115 human melanoma cell-line, we showed that RAGE overexpression resulted in increased cell migration and invasion, but decreased cell proliferation [79]. In the lung cancer cell-line H358, overexpression of RAGE was shown to reduce cell proliferation [80]. A study investigating several RAGE-expressing oral squamous cell carcinoma cell-lines showed that RAGE silencing affected variably the proliferation and invasion of these cell-lines. In this study, the authors showed that RAGE could stimulate proliferation, without affecting invasion, in the LMF4 and KKm oral squamous cell carcinoma cell-lines, and reciprocally, RAGE could stimulate invasion, without affecting cell proliferation, in the oral squamous cell carcinoma HSC3 and KKp cell-lines [81]. Distinct effects of RAGE on cell proliferation and migration were also observed in breast cancer cells and tumors. Kwak et al. showed that in MDA-MB-231 breast cancer cells, RAGE overexpression did not affect cell proliferation but resulted in increased cell migration as well as higher metastases formation when the cells were injected in mice [82]. Inversely, RAGE silencing of the highly metastatic breast cancer cell-line 4175 resulted in significant decreases in invasion as well as metastasis in mice. The diversity of reported effects of RAGE on cancer cell behavior makes it necessary to study individual cell types or cell-lines to gain an understanding of the role of RAGE for tumor development and progression. It appears that it is not possible to generalize the role of RAGE in cancer, and that indeed a detailed and differentiated understanding of RAGE in different tumor types and possible subtypes is necessary.

The available literature demonstrates that RAGE signaling is complex and depends on the cancer type, as well as on the type of ligand that activates RAGE. An example of ligand-dependent RAGE signaling was in colon cancer cells where AGEs increased cell migration and invasion predominantly through increased activities of iNOS and NF-κB, whereas in the same cells, HMGB1 acted mainly through activation of ERK1/2, Rac1, and Akt [83]. By contrast, in prostate cancer cells, RAGE activation by AGEs resulted in increased cell proliferation through the activation of PI3K, Akt, and the phosphorylation of retinoblastoma, resulting in the release and activation of the transcription factor E2F [84]. We have previously shown that in human WM115 melanoma cells, where RAGE overexpression stimulated cell migration and decreased cell proliferation, the activity of the MAP kinase ERK1/2 was decreased, but no changes in Akt or JNK activity were observed [79]. However, in these cells, the activity of the NF-κB transcription factor was also decreased [79]. In C6 glioma, RAGE promoted cell migration through the activation of Rac-1 and cdc42 [85]. In these cells, RAGE was shown to transduce its signal through the adaptor protein diaphanous-1 (Dia-1) that interacts with the cytoplasmic domain of RAGE. Dia-1, however, does not appear to be a universal adaptor protein for RAGE [86]. In RAGE transfect HEK293 cells, it was shown that the intracellular domain of RAGE interacted with two adaptor proteins other than Dia-1, TIRAP, and MyD88, in a ligand dependent manner as well. In this study, it was shown that RAGE engagement by HMGB1 resulted the in recruitment of these adaptor proteins whereas adaptor protein recruitment was not important when RAGE was activated by S100A8/A9 [86].

In the present study using Panc-1 cells, we demonstrate that the activity of ERK1/2, Akt, and NF-κB was decreased. Our data are thus in agreement with those from previously published studies and expand the understanding of the function of RAGE as a modulator of major signaling pathways in pancreatic cancer cells. Our data also suggest that additional studies, including animal studies, will be necessary to further evaluate the potential of RAGE as a drug target in pancreatic cancer.

## Figures and Tables

**Table 1:** Sequence of primers used in this study.

Primer Name	Sequence
β-Actin fwd	CATGTACGTTGCTATCCAGGC
β-Actin rev	CTCCTTAATGTCACGCACGAT
RAGE fwd	TGTGTGGCCACCCATTCCAG
RAGE rev	GCCCTCCAGTACTACTCTCG
α2 Integrin fwd	CCTACAATGTTGGTCTCCCAGA
α2 Integrin rev	AGTAACCAGTTGCCTTTTGGATT
β1 Integrin fwd	CCTACTTCTGCACGATGTGATG
β1 Integrin rev	CCTTTGCTACGGTTGGTTACATT

**Table 2:** List of primary antibodies and secondary antibodies used by Western blot (WB) and flow cytometry (FC).

Antigen Name (Poly- or Mono-Clonal)	Method (Dilution Used)	Cat. Number	Provider	Species
Akt (mono)	WB (1/1000)	4685	Cell Signaling	Rabbit
P-Akt (mono)	WB (1/2000)	4060	Cell Signaling	Rabbit
ERK1/2 (mono)	WB (1/1000)	4695	Cell Signaling	Rabbit
P-ERK1/2 (poly)	WB (1/1000)	9101	Cell Signaling	Rabbit
RAGE N-16 (poly)	WB (1/500)	sc-8230	Santa Cruz Biotechnologies	Goat
β-Actin (poly)	WB (1/500)	sc-1616	Santa Cruz Biotechnologies	Goat
FAK (mono)	WB (1/500)	sc-271126	Santa Cruz Biotechnologies	Mouse
P-FAK (mono)	WB (1/500)	sc-81493	Santa Cruz Biotechnologies	Mouse
Vimentin (mono)	WB (1/1000)	sc-6260	Santa Cruz Biotechnologies	Mouse
E-Cadherin (mono)	WB (1/10000)	ab40772	Abcam	Rabbit
N-Cadherin (mono)	WB (1/1000)	13116	Cell signaling	Rabbit
β-Catenin (mono)	WB (1/1000)	8480	Cell Signaling	Rabbit
ZEB1 (mono)	WB (1/1000)	3396	Cell Signaling	Rabbit
ZO-1 (mono)	WB (1/1000)	8193	Cell Signaling	Rabbit
Snail (mono)	WB (1/1000)	3879	Cell Signaling	Rabbit
Slug (mono)	WB (1/1000)	9585	Cell Signaling	Rabbit
β1 Integrin (mono)	WB (1/10000)	ab52971	Abcam	Rabbit
β1 Integrin (mono)	FC (1/100)	MA5-31981	Invitrogen	Rabbit
α2 Integrin (mono)	WB (1/10000)	ab133557	Abcam	Rabbit
α2 Integrin (mono)	FC (1/500)	EPR5788	Abcam	Rabbit
AlexaFluor 488 conjugated secondary antibody (poly)	FC (1/1000)	4412	Cell Signaling	Goat
HRP-conjugated donkey anti-goat antibody (poly)	WB (1/50000)	711-035-152	Jackson ImunoResearch	Donkey
HRP-conjugated goat anti-mouse antibody (poly)	WB (1/50000)	115-035-062	Jackson ImmunoResearch	Goat

## Abbreviations

RAGE	Receptor for advanced glycation endproducts
FLR2	Full-length RAGE Panc-1 clone 2
FLR3	Full-length RAGE Panc-1 clone 3
WT	Wild-type
